# Natural bioactive compounds form herbal medicine in Alzheimer’s disease: from the perspective of GSK-3β

**DOI:** 10.3389/fphar.2025.1497861

**Published:** 2025-02-07

**Authors:** Mei Wang, Wendi Huang, Juan Huang, Yong Luo, Nanqu Huang

**Affiliations:** ^1^ Department of Neurology, Third Affiliated Hospital of Zunyi Medical University (The First People’s Hospital of Zunyi), Zunyi, Guizhou, China; ^2^ Key Laboratory of Basic Pharmacology and Joint International Research Laboratory of Ethnomedicine of Ministry of Education, Zunyi Medical University, Zunyi, Guizhou, China; ^3^ Chinese Pharmacological Society-Guizhou Province Joint Laboratory for Pharmacology, Zunyi, Guizhou, China; ^4^ Department of Geriatrics, Third Affiliated Hospital of Zunyi Medical University (The First People’s Hospital of Zunyi), Zunyi, Guizhou, China; ^5^ National Drug Clinical Trial Institution, Third Affiliated Hospital of Zunyi Medical University (The First People’s Hospital of Zunyi), Zunyi, Guizhou, China

**Keywords:** Alzheimer’s disease, GSK-3β, tau protein, tau hyperphosphorylation, natural bioactive compounds

## Abstract

Alzheimer’s disease (AD) is a progressive neurodegenerative disorder characterized by cognitive decline and memory loss. Glycogen synthase kinase 3β (GSK-3β) plays a pivotal role in AD pathogenesis, particularly in tau protein hyperphosphorylation. Natural bioactive compounds have a wide range of sources, and medicinally valuable active compound can be extracted from plants, animals, and microorganisms. Currently, studies have found that various natural bioactive compounds from plants have the potential to improve AD symptoms, such as resveratrol and berberine. Therefore, this review examines the potential of natural bioactive compounds to modulate GSK-3β activity and inhibit the hyperphosphorylation of tau, offering a promising therapeutic strategy for AD. We summarize the current understanding of alkaloids, phenols, flavonoids, terpenoids and other natural compounds, highlighting their mechanisms of action and preclinical efficacy.

## Introduction

Alzheimer’s disease (AD) is the most prevalent neurodegenerative disorder, with an increasing prevalence observed on an annual basis. The decline in memory and cognitive functions in patients places a significant burden on their families and society. A principal pathological characteristic of AD is the hyperphosphorylation of tau proteins, which results in the formation of neurofibrillary tangles (NFTs) (Rostagno, 2022). In a healthy state, tau proteins function as microtubule-associated proteins, contributing to the maintenance of cellular structural stability (Shahani and Brandt, 2002). However, hyperphosphorylated tau proteins are detached from microtubules and accumulate in the brain, forming NFTs, a process that is closely related to the activity of Glycogen synthase kinase 3β (GSK-3β) (Bielska and Zondlo, 2006; Liu et al., 2023). GSK-3β is a multifunctional serine/threonine protein kinase that plays a pivotal role in regulating cellular function by participating in a multitude of signaling pathways. The activity of GSK-3β is subject to dual regulation, it is activated through auto-phosphorylation at tyrosine 216 and inactivated through phosphorylation at serine 9 (Krishnankutty et al., 2017). GSK-3β is capable of regulate the phosphorylation of multiple sites of the Tau proteins, including Thr181, Ser199, Ser202, and so forth (Liu et al., 2002). In the brains of AD patients, abnormal activation of GSK-3β is associated with the hyperphosphorylation of tau proteins and the formation of NFTs. Additionally, a decrease in the activity of Protein Phosphatase 2A (PP2A), the primary tau phosphatase, further exacerbates the imbalance of tau protein phosphorylation (Nicolia et al., 2010). Therefore, inhibiting the activity of GSK-3β is considered a potential therapeutic strategy for AD. Currently, a variety of GSK-3β inhibitors have been employed with some success in the evaluation of preclinical studies and experiments in AD (Arciniegas Ruiz and Eldar-Finkelman, 2021). However, the challenge of applying them to the clinic remains significant. This phenomenon can be attributed to the intricate nature of the pathogenesis of AD, and the likelihood of achieving therapeutic goals through a single mechanism is relatively low. Therefore, identifying drug with multi-target effects may prove to be a more efficacious approach for the treatment of AD.

Natural bioactive compounds widely distributed in plants, animals, marine organisms and microorganisms, and exhibit diverse chemical structures and a wide range of pharmacological activities. Researchers have used three-month-old male Albino Wistar rats to establish an AD model and have found that resveratrol, a naturally occurring compound derived from plants, has neuroprotective effects (Rao et al., 2024). Similarly, berberine, sourced from the plant *Coptis Salisb*, has demonstrated the ability to suppress the activation of GSK-3β and diminish the hyperphosphorylation of the tau protein in cellular models (Yu et al., 2011). Currently, the potential of natural bioactive compounds to treat AD by modulating GSK-3β activity is gradually being investigated (Huang et al., 2022; Santi et al., 2024; Xing et al., 2024). Thus, the aim of this work is to provide a comprehensive overview of *in vivo* and *in vitro* experiments investigating the regulation of Tau hyperphosphorylation by natural bioactive compounds through targeting GSK-3β (Table 1). This will facilitate the identification of novel therapeutic avenues for AD patients. Although GSK-3β inhibitors have shown some efficacy in preclinical studies, translating them into clinical applications remains challenging. Therefore, delving into the mechanisms of action and clinical application potential of these natural compounds is of great significance for the development of new AD therapeutic drugs.

**TABLE 1:** Natural bioactive compounds regulate the phosphorylation of Tau by affecting the activity of GSK-3β.

Metabolites	Source	Chemical structure	Mechanism	Test subject	Ref.
Berberine	*Coptis Salisb*	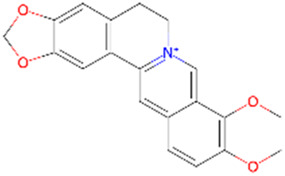	GSK-3β and CDK-5↓	Wistar rats with streptozotocin (STZ)	Saleh et al. (2024)
Gelsemine	*Gelsemium elegans Benth*	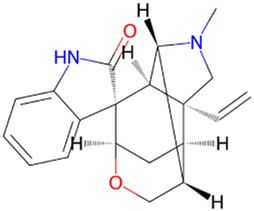	pSer9-GSK-3β↑	Aβ oligomer-treated mice	Chen et al. (2020)
Rutaecarpine	*Evodia rutaecarpa (Juss.) Benth*	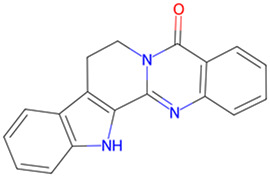	GSK-3β↓	C57BL/6 mice with high sucrose and pAAV-CMV-mGSK-3β	Zhao et al. (2021)
Dendrobium nobile Lindl. Alkaloid	*Dendrobium nobile Lindl*	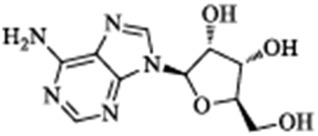	PI3K/Akt/GSK-3β pathway↑	Wortmannin (WM) and GF-109203X (GFX)-induced hyperphosphorylation of Tau in N2a cells and rats	Huang et al. (2024a)
Tetrahydroalstonine	*Cornus officinalis Sieb. et Zucc*	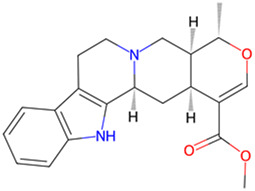	PI3K/Akt pathway↑, BACE1, GSK-3β, insulin resistance↓	Palmitate acid-induced SK-N-MC cells	Chen and Yu (2024)
Salidroside	*Rhodiola rosea L*	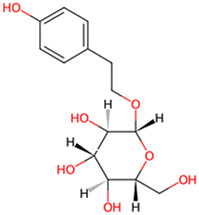	GSK-3β phosphorylation↑	Tau transgenic *Drosophila* line	Zhang et al. (2016)
Curcumin	*Curcuma longa L*	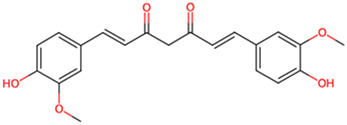	CDK-5 and GSK-3β↓	Scopolamine-induced AD rats	Das et al. (2019)
Resveratrol	Fruits	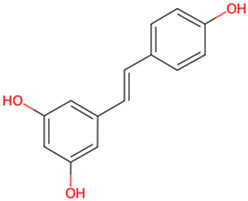	GSK-3β and ERK1/2↓	Hippocampal slice from 10-day-old Sprague–Dawley rat pup	Jhang et al. (2017)
Gallic acid	Medicinal plants	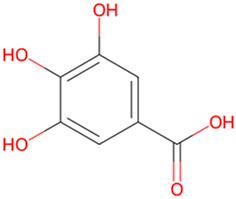	GSK-3β↓	APP/PS1 transgenic mouse	Ding et al. (2024)
Galangin	*Alpinia officinarum Hance*	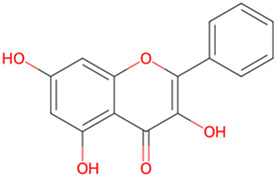	Akt/GSK3β/mTOR pathway↑	PC12 cells with Okadaic acid	Huang et al. (2019)
Scutellaria flavonoids	*Scutellaria Baicalensis Georgi*	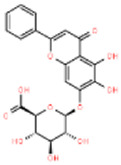	PKA↑, CDK-5 and GSK-3β↓	SD rats with Okadaic acid	Gao et al. (2021)
Icaritin	*Epimedium brevicornu Maxim*	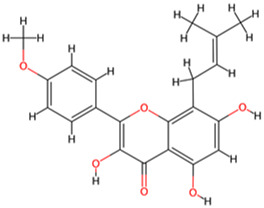	GSK-3β↓	SH-SY5Y cells with Okadaic acid	Li et al. (2022)
Icariin	*Epimedium brevicornu Maxim*	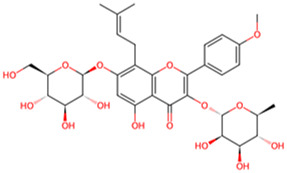	GSK-3β↓	SH-SY5Y cells with Okadaic acid	Li et al. (2022)
Genipin	*Gardenia jasminoides J. Ellis*	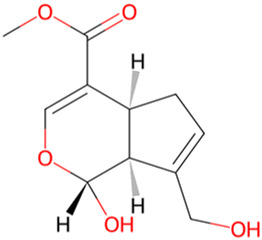	CDK-5 and GSK-3β↓	HEK293/Tau cells N2a/SweAPP cells SH-SY5Y/Tau cells	Li et al. (2021a)
Tanshinone IIA	*Salvia miltiorrhiza Bunge*	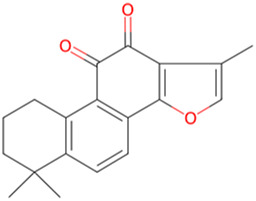	PI3K/Akt/GSK-3β pathway↑	APP/PS1 mice	Peng et al. (2022)
Ginsenoside Rd	*Panax ginseng* *C. A. Mey*	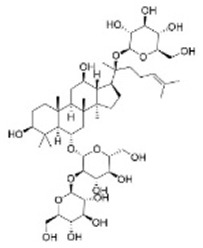	GSK-3β and CDK-5↓	APP transgenic mice	Li et al. (2021b)
Ginsenoside Rh4	*Panax ginseng* *C. A. Mey*	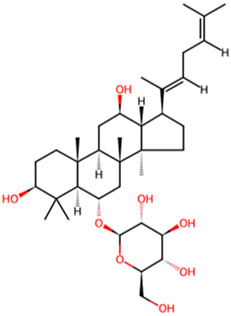	Wnt2b/GSK-3β/SMAD4 pathway↑	AD mouse induced by a combination of AlCl_3_·6H_2_O and d-galactose	Ren et al. (2023)
Atractylenolide III	*Atractylodes macrocephala Koidz*	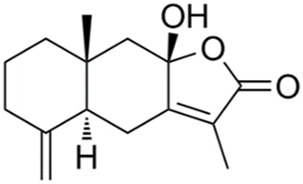	PI3K/Akt/GSK-3β pathway↑	SD rats through bilateral intracerebroventricular (ICV) administration of streptozotocin (STZ)	Liu et al. (2024)
Schisantherin B	*Schisandra chinensis (Turcz.) Baill*	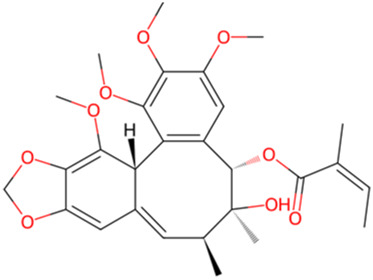	PI3K/Akt/GSK-3β pathway↑	Mice with Aβ_1-42_	Xu et al. (2016)
Sulforaphene	*Raphanus sativus L*	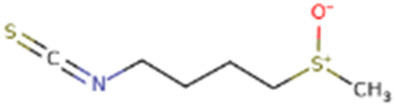	PI3K/Akt/GSK-3β Pathway↑	SD rats with Streptozotocin (STZ) and murine microglial BV-2 cell with lipopolysaccharide (LPS)	Yang et al. (2020)

The arrow ↑ indicates upregulation, and the arrow ↓ indicates downregulation.

## Alkaloids

Alkaloids are a class of nitrogen-containing organic bases primarily found in plants, known for their diverse physiological functions and biological activities. They typically have complex cyclic structures, with nitrogen atoms often included within the rings. Alkaloids have a broad range of pharmacological effects, including anti-cancer, anti-bacterial, anti-inflammatory, and antioxidant properties. Moreover, alkaloids also play a significant role in the treatment of neurological diseases.

Berberine, an isoquinoline alkaloid, is extracted from the *Coptis Salisb*, which is classified within the Ranunculaceae species. In the present study, researchers utilized berberine (BBR)-loaded poly (lactic-co-glycolic acid) (PLGA)/Tet-1 peptide nanoparticles (BBR/PLGA-Tet NPs) to evaluate the therapeutic potential of BBR in a rat AD model induced by streptozotocin (STZ). The findings revealed that both BBR and BBR/PLGA-Tet NPs significantly ameliorated cognitive impairments induced by STZ in AD rats, with BBR/PLGA-Tet NPs demonstrating a more pronounced effect. This improvement may be attributed to the reduction of GSK-3β and CDK-5 protein levels, thereby decreasing the hyperphosphorylation of Tau (Saleh et al., 2024). These nanoparticles can effectively penetrate the blood-brain barrier, enhancing the delivery efficiency of the drug within the brain, thereby providing a new strategy for the treatment of AD. This provides an experimental basis for the treatment of AD. Gelsemine, derived from *Gelsemium elegans Benth.*, which is classified within the Loganiaceae. It possesses not only anti-inflammatory and antioxidant effects but also inhibits the production of inflammatory factors. In a mouse model of β-amyloid (Aβ) oligomer-induced AD, Gelsemine demonstrated significant activity at a dose of 5 μg/kg. This activity was observed to reduce cognitive deficits and inflammatory responses induced by Aβ oligomers, as well as augment the phosphorylation level of GSK-3β at the Ser9 site. Consequently, this resulted in a reduction in the hyperphosphorylation of Tau, producing improved AD (Chen et al., 2020). However, this study injected the drug directly into the mouse brain via a stereotaxic device, a risky method of administration that is not directly applicable to human experimentation. Additionally, it is challenging to ascertain the accuracy of the drug injection into specific brain regions, owing to technological limitations. Another alkaloid, rutaecarpine, is extracted from the plant *Evodia rutaecarpa (Juss.) Benth.*, which is classified within the *Rutoideae*. Zhao et al. constructed an AD model using an adeno-associated virus carrying the GSK-3β gene (pAAV-CMV-mGSK-3β), which was injected by stereotaxic injection into the brains of mice. These mice had been fed a 20% sucrose solution and a 0.01% rutaecarpine chow for 24 weeks. They found that rutaecarpine at 0.01% was found to show significant pharmacological activity, ameliorating spatial memory deficits and enhancing synaptic plasticity in AD mice by modulating the GSK-3β signaling pathway (Zhao et al., 2021). However, this experimental method is not rigorous. Feeding through diet cannot guarantee that each mouse ingests the same amount of the drug. This may weaken the persuasiveness of the experimental results. Administering a fixed drug concentration via gavage to simulate oral drug intake in humans may better illustrate the pharmacological effects of the drug and reduce experimental errors. Dendrobium nobile Lindl. Alkaloid (DNLA), found in the valuable Orchidaceae species *Dendrobium nobile Lindl*. In experiments conducted *in vivo* and *in vitro*, Our previous research found that DNLA (20 mg/kg) could effectively reverse the hyperphosphorylation of the Tau protein in N2a cells and Wistar rats by regulating the PI3K/Akt/GSK-3β signaling pathway (Huang J. et al., 2024). Furthermore, tetrahydroalstonine (THA), another active compound extracted from Cornaceae species *Cornus officinalis Sieb. et Zucc*. In an *in vitro* experiment, THA (10 μM) activates the impaired PI3K/AKT signaling pathway, regulating insulin resistance and inhibiting the activity of BACE1 and GSK-3β, leading to a reduction in the production of Tau and Aβ (Chen and Yu, 2024). These findings indicate that some alkaloids, by modulating the activity of GSK-3β, have a significant inhibitory effect on the hyperphosphorylation of tau protein, offering new strategies for the treatment of AD.

## Phenols

Phenols are widely present in a variety of plants found in nature, such as fruits, tea leaves, grains, and vegetables. These compounds not only demonstrate potential health benefits in preventing cardiovascular diseases, inflammation, tumors, bacteria, and viruses, but some also exhibit neuroprotective effects, showing promise in improving symptoms of AD. Salidroside, extracted from *Rhodiola rosea L*, Crassulaceae family, has been demonstrated to alleviate the hyperphosphorylation of tau protein in a transgenic fruit fly model of AD. Using donepezil as a positive control group, the researchers ascertained that 2 μM of salidroside prolonged lifespan and enhanced locomotor activity in tau transgenic *Drosophila*, thereby demonstrating its potential for the treatment of AD. This effect was achieved by enhancing the phosphorylation of GSK-3β (Zhang et al., 2016). Curcumin, a naturally occurring yellow pigment derived from Zingiberaceae species *Curcuma longa L*, reverses spatial memory and motor deficits in scopolamine-induced AD rats by inhibiting the activity of GSK-3β and Cyclin dependent Kinase 5 (CDK-5), reducing the aggregation of Aβ and hyperphosphorylation of tau (Das et al., 2019). In this study, curcumin (80 mg/kg) demonstrated the same therapeutic effect as donepezil, with potential to treat AD. Resveratrol (RES), a polyphenolic compound with antioxidant, anti-inflammatory, and antimicrobial properties. Researchers utilized hippocampal slices from Sprague-Dawley rats to conduct their study. Initially, they increased the levels of p-S396-tau in the hippocampal slices through the application of Na3VO4. Subsequently, they intervened with resveratrol for a duration of 1 hour. It was discovered that RES (at a concentration of 20 μM) significantly enhanced the phosphorylation of GSK-3β at the Ser9 site, thereby inhibiting the activity of GSK-3β. This action led to a reduction in the Na3VO4-induced levels of p-S396-tau. Additionally, RES also suppressed the activation of ERK1/2 induced by Na3VO4, demonstrating its potential therapeutic role in AD (Jhang et al., 2017). The study was the first to reveal the connection between the generation of reactive oxygen species (ROS) caused by long-term exposure to Na3VO4 and the phosphorylation of tau protein, providing new insights into the pathological mechanisms of AD. Through the use of various drug pre-treatments and long-term exposure experiments, the protective effects of resveratrol were systematically evaluated. However, the sample size was relatively small (n = 3–5), which may increase the likelihood of randomness in the results, thereby affecting the reliability of the conclusions. Gallic acid (GA), isolated from medicinal plants, has been shown to significantly reduce abnormal phosphorylation levels of tau protein and the accumulation of Aβ in the APP/PS1 transgenic mouse model, thereby ameliorating spatial memory deficits in AD model mice. This effect is attributed to the interaction of GA with key phosphorylation sites of GSK-3β, thereby inhibiting its activity (Ding et al., 2024). These studies indicate that some phenols compounds show potential in the treatment of AD by inhibiting the activity of GSK-3β and reducing the hyperphosphorylation of tau, offering new strategies for improving memory and cognitive functions in patients with AD. Future research should further explore the mechanisms of action and clinical application possibilities of these compounds.

## Flavonoids

Flavonoids are a class of secondary compounds found in plants. They are widely distributed in plant parts such as flowers, leaves, stems, and fruits. They are named for their yellow pigment properties. The pharmacological effects of these compounds have been confirmed by scientific research, which has demonstrated that they possess anti-inflammatory, antioxidant, antibacterial, and antiviral properties. In this article, we will present three natural compounds that have the potential to be used in the treatment of AD. Galangin is a bioactive compound that is extracted from Zingiberaceae family *Alpinia officinarum Hance*. In the PC12 cell model of AD, Galangin (1.0 μg/mL) has been observed to enhance cell viability and reduce tau protein phosphorylation by modulating the Akt/GSK-3β/mTOR signaling pathway, thereby inhibiting the activity of GSK-3β (Huang et al., 2019). Although the authors found that it has certain therapeutic effects on AD *in vitro* models, there is a lack of further verification *in vivo* models. There are certain differences between the *in vivo* environment and *in vitro* experiments. Conducting further animal experiments may better demonstrate the potential value of this compound. Moreover, stem and leaf flavonoids from *Scutellaria Baicalensis Georgi* (SSF), which is member of the Labiatae family, have been found to enhance learning and memory capabilities in AD rats by regulating the activity of CDK-5, PKA, and GSK-3β, which in turn inhibits the hyperphosphorylation of tau protein (Gao et al., 2021). Icaritin (ICT) and icariin (ICA), extracted from the Chinese botanical drug *Epimedium brevicornu Maxim*, Berberidaceae family. Our research found that 2.5 μmol/L of ICA and 1.0 μmol/L of ICT significantly reduced the levels of p-Tau and GSK-3β in the SH-SY5Y cell model induced by okadaic acid (OA), highlighting its neuroprotective potential, and that ICT was slightly more effective than ICA (Li et al., 2022). These evidences indicate that flavonoids have the potential to be a valuable therapeutic option for AD and further investigation into their neuroprotective capabilities is warranted. It will be instrumental in assessing the viability of flavonoids as therapeutic agents for the mitigation of AD pathology and related neurodegenerative processes.

## Terpenoids

Terpenoids constitute a class of natural hydrocarbon compounds that are widely distributed in plants and animals. They can be categorized based on the number of isoprene units they contain, resulting in the following classifications: monoterpenes (C_10_H_16_), sesquiterpenes (C_15_H_24_), diterpenoids (C_20_H_32_), triterpenoids (C_30_H_48_), and tetraterpenes (C_40_H_64_). A number of terpenoids have been found to possess biological activities, including antimalarial, anticancer, anti-inflammatory and antiviral properties. Additionally, the neuroprotective effects of terpenes are being increasingly investigated. Genipin is a bioactive compound that is extracted from *Gardenia jasminoides J. Ellis,* Rubiaceae family. The researchers observed the cell physiological changes after Genipin treatment at different concentrations in a variety of cell lines, and found that Genipin (20 μM) could inhibit Tau phosphorylation by down-regulating the expression of CDK-5 and GSK-3β. Genipin (20 μM) was found to inhibit Tau phosphorylation by down-regulating the expression of CDK-5 and GSK-3β, and to activate mTOR-dependent autophagy through the SIRT1/LKB1/AMPK signaling pathway, while inhibiting Aβ production, thus exerting neuroprotective effects (Li M. et al., 2021). Tanshinone IIA (TanIIA), extracted from the Chinese botanical drug Labiatae species *Salvia miltiorrhiza Bunge.* In the APP/PS1 mouse model of AD, following a 4-week period of TanIIA treatment, researchers observed the activation of the PI3K/Akt signaling pathway and inhibition of GSK-3β. This resulted in a significant attenuation of Tau hyperphosphorylation, as well as the reversal of cholinergic dysfunction and the reduction of oxidative stress. Notably, the low-dose group (15 mg/kg) and the high-dose group (30 mg/kg) exhibited comparable outcomes (Peng et al., 2022). However, it should be noted that the findings may be affected by the limited sample size, which could potentially compromise the representativeness and statistical validity of the results. Araliaceae species *Panax ginseng C. A. Mey* has been used for thousands of years in China. In one study, ginsenoside Rd effectively decreased the production and deposition of hyperphosphorylated tau protein by depressing the expression of GSK-3β and CDK-5 (Li L. et al., 2021). Further research has found that ginsenoside Rh4, which has higher pharmacological activity than ordinary ginsenosides, can not only inhibit the inflammatory response caused by over-activation of microglia and astrocytes, but also inhibit the excessive phosphorylation of tau protein in the hippocampus of AD mouse by regulating the Wnt2b/GSK-3β/SMAD4 signaling pathway (Ren et al., 2023). Atractylenolide III (Liu et al., 2024) extracted from *Atractylodes macrocephala Koidz*, Compositae family, has been demonstrated to enhance learning and memory capabilities in AD rats through modulate the PI3K/AKT/GSK-3β signaling pathway, and it has the same effect as donepezil. The results of these studies indicate that specific terpenoids have the potential to modulate the aberrant phosphorylation and aggregation of tau protein by regulating the phosphorylation of GSK-3β. In light of these findings, there is a scientific rationale for further investigation and development of terpenes as potential therapeutic strategies for AD.

## Others

In addition to the natural bioactive compounds mentioned above, there are other classes of compounds found in nature that can exert neuroprotective effects by modulating key cellular signaling pathways. Schisantherin B is a phenylpropanoid compound extracted from the plant *Schisandra chinensis (Turcz.) Baill.* of the Magnoliaceae family. In AD mouse model, Schisantherin B at a dose of 0.15 mg/kg has demonstrated the ability to reduce excessive phosphorylation of Tau, which may be achieved by modulating GSK-3β (Xu et al., 2016). Although this study used donepezil as a positive control group, which indicates the therapeutic potential of Schisantherin B. However, the specific mechanism of action was not thoroughly investigated, and there is a lack of research on the biological toxicity of this compound. Future studies can include additional experimental content to further explore its molecular mechanisms. Sulforaphene (SF) represents a primary isothiocyanate compound that has been extracted from the Cruciferae species *Raphanus sativus L*. In the AD rat model, oral administration of SF (25 and 50 mg/kg) over a period of 6 weeks resulted in a significant improvement in cognitive function in rats. In addition, researchers conducted cell experiments and found that SF has potential anti-inflammatory effects in LPS-induced BV-2 cells. This may be achieved by regulating the PI3K/Akt/GSK-3β signaling pathway (Yang et al., 2020). Despite their disparate origins, these compounds share a common mechanism of action, namely, the inhibition of GSK-3β activity and the reduction of tau protein phosphorylation. These findings indicate the potential of natural products in the treatment of neurodegenerative diseases and provide a scientific basis for the development of new therapeutic drugs.

## Conclusion

**FIGURE 1:**
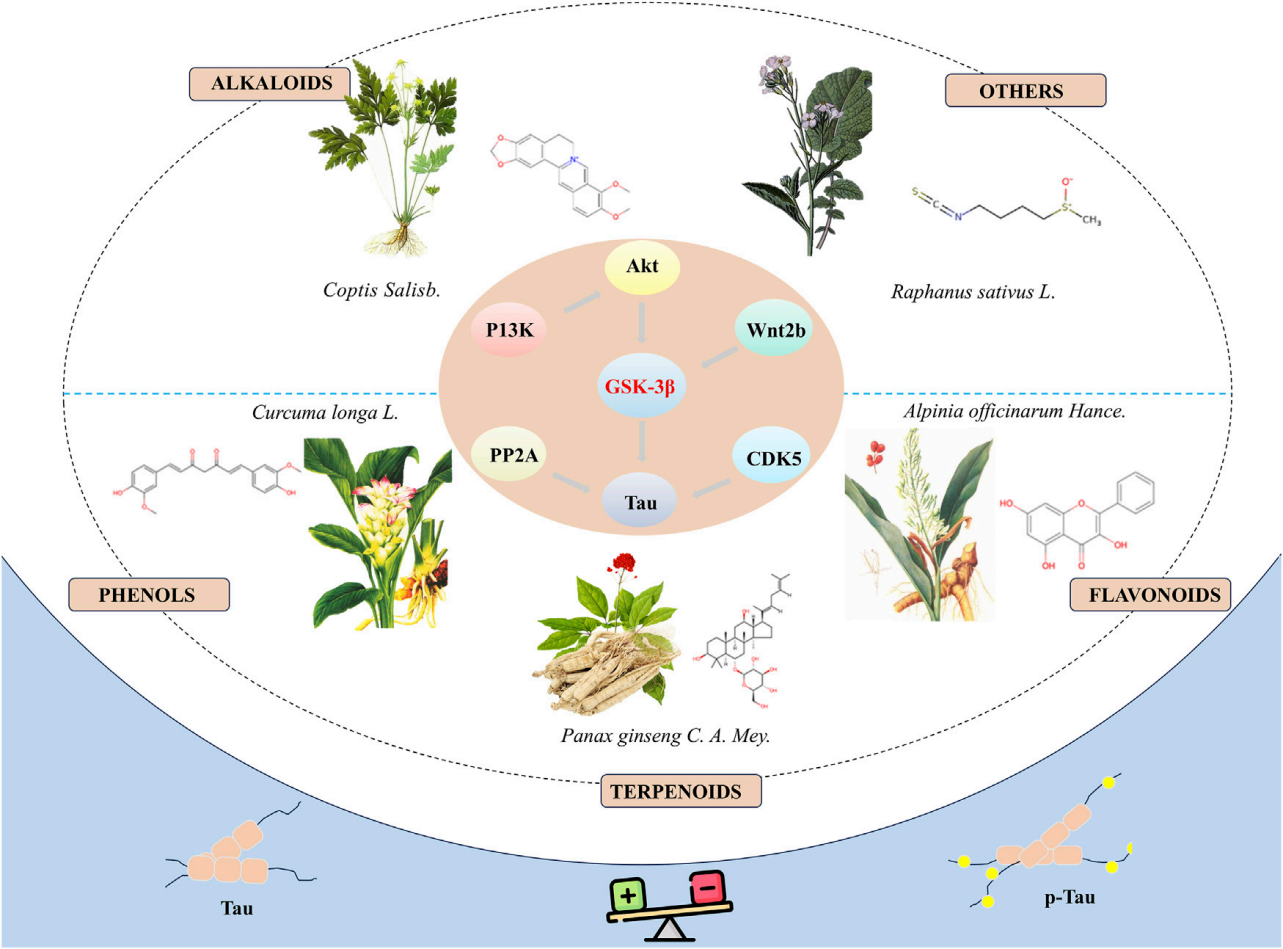
Natural bioactive compounds inhibit the hyperphosphorylation of tau protein by modulate the activity of GSK-3β and demonstrate the potential to treat AD.

In summary, natural bioactive compounds have demonstrated the potential to inhibit the hyperphosphorylation of tau protein by directly or indirectly modulating the activity of GSK-3β, thereby exhibiting potential therapeutic effects in neuroprotection (Figure 1). Although these bioactive molecules show promise in the prevention and treatment of neurodegenerative diseases, there are several limitations in current research: (1) Most studies are based on *in vitro* cell models and animal experiments, lacking relevant clinical trials to further verify the exact efficacy and side effects of these compounds. (2) The specific molecular mechanisms through which these bioactive molecules regulate the activity of GSK-3β remain to be fully elucidated and require further elucidation in future research. The broad role of GSK-3β suggests that its inhibition may have therapeutic benefits for a range of diseases, including diabetes (Lanzillotta et al., 2024) and cancer (Furuta et al., 2017). This extensive therapeutic potential provides opportunities for drug development but also increases the risks associated with drug use, as the role of GSK-3β can be completely different and even contradictory in different disease states. For instance, in certain tumour types, GSK-3β may act as a tumour suppressor, while in other tumour types, it may act as a tumour promoter (Luan et al., 2024). (3) Existing research has primarily focused on the short-term effects of these drugs, with a relative lack of systematic assessment of long-term efficacy and safety. (4) The reliability and generalizability of results may be affected by limitations in sample size or flaws in study design. The majority of experiments only utilize negative controls, which can exclude the influence of certain factors in the experiment, but cannot fully demonstrate that these compounds are more effective than currently used clinical drugs. (5) Although some compounds have shown promising results in cellular models, their toxicity and side effects *in vivo* are still unclear, necessitating further evaluation through more animal experiments and clinical trials. (6) Plant extracts are complex mixtures with compositions that vary depending on the preparation methods and the plant materials used. This complexity and variability impact the reproducibility and interpretation of research. To address these challenges, some guidelines for the classification of plant extract studies can be used to improve the reproducibility and interpretability of research (Heinrich et al., 2022). (7) Many of these compounds may suffer from poor absorption, rapid metabolism, or inadequate ability to cross the blood-brain barrier, which can significantly diminish their therapeutic effectiveness *in vivo*. Combining new materials to solve the problem of drug delivery is also a valuable research direction (Wu et al., 2023). Consequently, in subsequent research, it is essential to devise more comprehensive experimental plans and to explore related mechanisms in depth.

AD is a complex neurodegenerative disorder with limited treatment options. It is for this reason that researchers are committed to discovering more effective and safe treatment options. The unique chemical structures and multi-target mechanisms of action of natural bioactive compounds offer new avenues for the treatment of AD. Despite the current limitations in research, future clinical trials are expected to further verify the efficacy and safety of these compounds in AD patients, potentially leading to new breakthroughs in AD therapy. Future research could consider combining multiple models to more comprehensively assess the effects of compounds. For instance, integrating network pharmacology with *in vitro* and *in vivo* models can leverage the strengths of these models to provide more convincing scientific evidence. Utilizing novel technologies such as organoid construction (Qian et al., 2017) and human cell models can more accurately simulate the *in vivo* environment of the human body, allowing for a deeper exploration of the potential adverse effects of these compounds on humans, thereby laying the foundation for subsequent clinical trials. And the development of artificial intelligence and large language models may bring new ideas and perspectives to this type of research (Huang N. et al., 2024). We should embrace new technologies with a more inclusive attitude.
